# An orthotopic xenograft model for high-risk non-muscle invasive bladder cancer in mice: influence of mouse strain, tumor cell count, dwell time and bladder pretreatment

**DOI:** 10.1186/s12885-017-3778-3

**Published:** 2017-11-23

**Authors:** Doreen Huebner, Christiane Rieger, Ralf Bergmann, Martin Ullrich, Sebastian Meister, Marieta Toma, Ralf Wiedemuth, Achim Temme, Vladimir Novotny, Manfred P. Wirth, Michael Bachmann, Jens Pietzsch, Susanne Fuessel

**Affiliations:** 1 Department of Urology, University Hospital Carl Gustav Carus, Technische Universität Dresden, Fetscherstrasse 74, 01307 Dresden, Germany; 2 Department Radiopharmaceutical and Chemical Biology, Helmholtz-Zentrum Dresden-Rossendorf (HZDR), Institute of Radiopharmaceutical Cancer Research, Bautzner Landstrasse 400, 01328 Dresden, Germany; 3 Institute of Pathology, University Hospital Carl Gustav Carus, Technische Universität Dresden, Fetscherstrasse 74, 01307 Dresden, Germany; 4 Department of Neurosurgery, Section Experimental Neurosurgery & Tumor Immunology, University Hospital Carl Gustav Carus, Technische Universität Dresden, Fetscherstrasse 74, 01307 Dresden, Germany; 5 German Cancer Consortium (DKTK), partner site Dresden, Germany, and German Cancer Research Center (DKFZ), Fetscherstrasse 74, 01307 Dresden, Germany; 6 National Center for Tumor Diseases (NCT) Dresden, University Hospital Carl Gustav Carus, Technische Universität Dresden, Fetscherstrasse 74, 01307 Dresden, Germany; 7 UniversityCancerCenter (UCC), University Hospital Carl Gustav Carus, Technische Universität Dresden, Fetscherstrasse 74, 01307 Dresden, Germany; 8 Department of Chemistry and Food Chemistry, School of Science, Technische Universität Dresden, Mommsenstrasse 4, 01069 Dresden, Germany

**Keywords:** Bioluminescence, Luciferase, Orthotopic xenograft models, Small animal multimodal imaging, Magnetic resonance imaging, Optical imaging, Positron emission tomography, Transurethral instillation, UM-UC-3 cell line, Urothelial carcinoma

## Abstract

**Background:**

Novel theranostic options for high-risk non-muscle invasive bladder cancer are urgently needed. This requires a thorough evaluation of experimental approaches in animal models best possibly reflecting human disease before entering clinical studies. Although several bladder cancer xenograft models were used in the literature, the establishment of an orthotopic bladder cancer model in mice remains challenging.

**Methods:**

Luciferase-transduced UM-UC-3^LUC^K1 bladder cancer cells were instilled transurethrally via 24G permanent venous catheters into athymic NMRI and BALB/c nude mice as well as into SCID-beige mice. Besides the mouse strain, the pretreatment of the bladder wall (trypsin or poly-L-lysine), tumor cell count (0.5 × 10^6^–5.0 × 10^6^) and tumor cell dwell time in the murine bladder (30 min – 2 h) were varied. Tumors were morphologically and functionally visualized using bioluminescence imaging (BLI), magnetic resonance imaging (MRI), and positron emission tomography (PET).

**Results:**

Immunodeficiency of the mouse strains was the most important factor influencing cancer cell engraftment, whereas modifying cell count and instillation time allowed fine-tuning of the BLI signal start and duration – both representing the possible treatment period for the evaluation of new therapeutics. Best orthotopic tumor growth was achieved by transurethral instillation of 1.0 × 10^6^ UM-UC-3^LUC^K1 bladder cancer cells into SCID-beige mice for 2 h after bladder pretreatment with poly-L-lysine. A pilot PET experiment using ^68^Ga-cetuximab as transurethrally administered radiotracer revealed functional expression of epidermal growth factor receptor as representative molecular characteristic of engrafted cancer cells in the bladder.

**Conclusions:**

With the optimized protocol in SCID-beige mice an applicable and reliable model of high-risk non-muscle invasive bladder cancer for the development of novel theranostic approaches was established.

## Background

Worldwide, bladder cancer (BCa) is the 9th most common cause of tumor-related death with estimated 429,000 new cases and 165,000 deaths in the year 2012 [1]. In Germany, about 30,000 people develop a BCa and approximately 6000 die of BCa each year [2]. Around 75% of newly diagnosed patients present with non-muscle invasive BCa (NMIBC) that is confined to the mucosa (stage Ta and *carcinoma* in situ) or submucosa (stage T1). Standard therapy for these patients is transurethral resection with adjuvant intravesical chemo- or immunotherapy [3]. Despite these therapies 21% of patients with high-risk NMIBC – for example patients with tumor stage T1 and/or high grade (= G3) tumors – progress to muscle invasive BCa and 14% die of BCa mainly within 4 years [4]. Therefore, alternative treatment options are needed which require thorough evaluation in preclinical models – first in cell culture and thereafter in animal models.

Most often mice are used in animal models because of their relatively high genetic homology to humans, their fast breeding cycle as well as the low costs for housing and maintenance [5]. An orthotopic xenograft model in which the human cancer is grown in the urinary bladder of the animal reflects the human counterpart, facilitates the evaluation of experimental therapeutics which require human cells (for example agents based on gene silencing) and allows intravesical application of experimental therapeutics which is the administration route used in NMIBC patients. If cancer cells which carry a bioluminescent or fluorescent reporter gene are used, monitoring of tumor growth is possible by non-invasive bioluminescence (BLI) or fluorescence imaging [6, 7]. A suitable orthotopic BCa xenograft model should (i) have a high rate of tumor cell engraftment, (ii) be reproducible and (iii) offer an appropriate treatment period with a well-defined therapy start. The utilization of human cancer cells requires the use of immunodeficient mice. Therefore, it is not possible to evaluate immune response of experimental therapeutics with such xenograft models. For the successful engraftment of tumor cells in the bladder it is essential to rupture the glycosaminoglycan layer which lines the mucosa and protects it from irritants and bacteria in the urine. Different mechanical (e.g. scraping with stylet or electrocautery) and chemical approaches (e.g. instillation of acid, trypsin or poly-L-lysine [PLL]) for overcoming the glycosaminoglycan layer are described (summarized in [8, 9]). Further factors which influence tumor incidence are for example the aggressiveness of the cancer cells, tumor cell count and dwell time of the cancer cells in the bladder. Rates of tumor engraftment increase with higher tumor cell numbers and prolonged incubation time [9].

Although, several BCa xenograft models have been described in literature, the establishment of an orthotopic model in mice remains challenging and rates of tumor cell engraftment vary from 67 to 80% if human BCa cells were instilled transurethrally using 22-G or 24-G catheters [10–12]. In these studies, the bladder wall was treated either with trypsin or PLL prior to tumor cell instillation to improve adherence of cells. Bladder pretreatment with electrocautery caused tumor formation in 80% of mice [13]. The implantation of cancer cells by percutaneous injection under ultrasound guidance revealed 100% tumor cell engraftment but all these cancers grew invasively [14]. In our study, we aimed at generating an orthotopic mouse model with luciferase-expressing human UM-UC-3 BCa cells as a model for high-risk NMIBC and examined the use of different immunodeficient mouse strains as well as the modification of tumor cell count, dwell time and pretreatment of bladder wall. Dedicated small animal BLI and magnetic resonance imaging (MRI) were performed in order to visualize successful cancer cell engraftment. A pilot positron emission tomography (PET) experiment with radiolabeled cetuximab was performed in order to characterize epidermal growth factor receptor (EGFR) expression as functional characteristic of engrafted UM-UC-3 tumors [15]. In this regard, EGFR exemplarily reflects a potential molecular target for (radio)immunotherapeutic treatment of BCa. Staging and grading of the orthotopic tumors as well as the formation of metastases were also determined.

## Methods

### Cell culture, viral transduction and generation of stable cell lines

The human BCa cell line UM-UC-3 (ATCC CRL-1749; ATCC, Rockville, MD, USA; bought in 2011) was cultured in minimum essential medium with 10% fetal calf serum and 1% non-essential amino acids (all from Life Technologies, Karlsruhe, Germany). Cells were cultured at 37 °C in a humidified atmosphere containing 5% CO_2_. To enable non-invasive visualization of tumor growth, UM-UC-3 cells were transduced with a retroviral pRevCMV-c-Luc vector containing the firefly luciferase gene (LUC+) [16] and a hygromycin B resistance cassette [17]. Packaging of viral particles and transductions were performed as previously described [18]. Afterwards, single UM-UC-3^LUC^ cells were seeded into 96-well plates, cultured and selected by adding 300 μg/ml hygromycin B to the culture medium. Depending on the growth and apoptosis pattern as well as on the strength of the luminescence signal the UM-UC-3^LUC^K1 clone was chosen for experiments.

### In vitro measurement of luciferase activity

For monitoring the cells, in vitro luciferase activity was measured regularly with Luciferase Assay System according to the manufacturer’s instructions (Promega, Mannheim, Germany). Furthermore, luciferase activity was measured after pouring different cell counts into a 96-well flat clear bottom black polystyrene TC-treated microplate using the In-Vivo Xtreme imaging system (Bruker BioSpin MRI GmbH, Ettlingen, Germany). In doing so, at least 2 wells were left blank between the measuring points. Five microliters D-luciferin (15 mg/ml in PBS; PerkinElmer, Rodgau, Germany) were added to 200 μl cell solution directly before imaging.

### Western blot analysis

Protein separation and subsequent Western blotting were performed as described previously [19]. Membranes were probed with primary antibodies against EGFR (1:1000; EGF Receptor Antibody #2232; Cell Signaling, Danvers, MA, USA) and β-actin (1:50,000; clone AC-74; Sigma, St. Louis, Missouri, USA); the latter served as a loading control. The secondary polyclonal swine anti-rabbit immunoglobulin HRP-linked antibody (1:1000; P0217; Dako Deutschland GmbH, Hamburg, Germany) as well as the Enhanced Chemiluminescence Kit (GE Healthcare, Freiburg, Germany) were used for visualization.

### Orthotopic xenograft model of human bladder cancer

The following immunocompromised mouse strains were used in the study: athymic NMRI nude (NMRI-*Foxn1* ^*nu*^ */Foxn1* ^*nu*^; Charles River Laboratories, Sulzfeld, Germany), BALB/c nude (*BALB/cAnNRj-Foxn1* ^*nu*^; Janvier Labs, Saint-Berthevin Cedex, France) as well as SCID-beige (*CB17.Cg-Prkdc* ^*scid*^ *Lyst* ^*bg-J*^ */Crl*; Charles River Laboratories). All three mouse strains lack T cells. In contrast to the other two mouse strains, SCID-beige mice also lack B cells, have impaired natural killer cell activity and are not hairless. General anesthesia was induced with 10% (*v/v*) and maintained with inhalation of 8% (*v/v*) desflurane (Suprane; Baxter, Unterschleissheim, Germany) in 30/10% (*v/v*) oxygen/air. For tumor cell instillation, fourteen weeks old female mice were used. Mouse bladders were catheterized using 24G permanent venous catheters (Becton Dickinson, Heidelberg, Germany) that were coated with petroleum jelly (Bombastus-Werke AG, Freital, Germany). To prevent bladder overexpansion residual urine was removed by massaging the bladder with thumb and trigger finger. UM-UC-3^LUC^K1 BCa cells were harvested, resuspended in PBS and vital cell count was determined using the cell counting system CASY model TT (Schaerfe System, Reutlingen, Germany). The desired cell count was adjusted in a total volume of 100 μl and cells were instilled into the urinary bladders. Pretreatment of the bladder wall was performed by incubating either 100 μl of 0.1 mg/ml poly-L-lysine (PLL, Sigma-Aldrich, Steinheim, Germany) for 20 min or 100 μl 0.5% trypsin in 0.2% EDTA (Sigma-Aldrich) for 30 min. Detailed conditions for the different experiments performed are listed in Table 1. General condition of the mice was determined every day and mouse weights twice a week. Necropsy was performed in dependence on luminescence intensity and occurrence of blood in urine as well as at reduced general conditions. Whole bladders were removed for histologic examinations. Additionally, kidneys, livers and lungs of all mice were removed in experiment 6.

**Table 1:** Summary of series of experiments for establishment of an orthotopic bladder cancer model in mice

	Experimental number					
	1	2	3	4	5	6
Mouse strain	NMRI nude	NMRI nude	**A: BALB/c nude** **B: SCID-beige**	**A: BALB/c nude** **B: SCID-beige**	SCID-beige	SCID-beige
Mice used (n)	12	18	20	20	20	16
Anaesthesia-related deaths (n)	2	0	1	1	1	0
Mice per treatment arm (n)	A: 5 B: 5	A: 9 B: 9	A: 10 B: 9	A: 10 B: 9	A: 9 B: 10	A: 8 B: 8
Cell count	2.0 × 10^6^	2.0 × 10^6^	2.0 × 10^6^	**A: 5.0** × **10** ^**6**^ **B: 1.0** × **10** ^**6**^	**A: 0.5** × **10** ^**6**^ **B: 1.0** × **10** ^**6**^	0.5 × 10^6^
Dwell time	2 h	2 h	2 h	2 h	1 h	30 min
Pretreatment of bladder	**A: trypsin** ^**a**^ **B: PLL** ^**a**^	**A: PLL** **B: PLL** ^**a**^	PLL	PLL	PLL	**A: PLL** **B: PLL** ^**b**^
Tumor cell engraftment	A: 2/5 (40%) B: 2/5 (40%)	A: 2/9 (22%) B: 3/9 (33%)	A: 7/10 (70%) B: 9/9 (100%)	A: 9/10 (90%) B: 9/9 (100%)	A: 9/9 (100%) B: 10/10 (100%)	A: 7/8 (88%) B: 8/8 (100%)
Signal start (d)	17.0 ± 2.0 7.5 ± 4.5	12.5 ± 5.5 19.7 ± 2.9	33.0 ± 14.3 16.7 ± 4.3	33.9 ± 18.3 14.8 ± 2.1	25.8 ± 3.5 22.4 ± 5.6	22.4 ± 2.9 19.4 ± 2.9
Signal duration (d)	18.0 ± 2.0 20.0 ± 3.0	12.0 ± 1.0 16.0 ± 6.7	14.0 ± 4.0 8.7 ± 3.0	10.6 ± 4.0 10.4 ± 1.4	13.3 ± 6.1 11.5 ± 4.6	19.6 ± 8.2 18.1 ± 8.9

Bold statements highlight the parameters that were varied in the experiment. Signal start and signal duration are shown as mean ± mean deviation

*Abbreviation*: *PLL* poly-L-lysine

^a^ Induction of lesions in the urothel by carefully scratching with the cannula of the permanent venous catheters

^b^ To avoid air bubbles in the bladder the catheter was filled with cell suspension prior to catheterization

### Histology

Tissues were fixed in 4% buffered formalin, embedded in paraffin and cut in 3 μm sections which were stained with haematoxylin and eosin (H&E) using standard techniques. All slides were reviewed by an experienced pathologist. T stage was assessed according to 7th edition of TNM Classification of Malignant Tumours [20].

### Small animal imaging of tumor xenograft models

Multimodal imaging of tumor growth (BLI, MRI) and functional characteristics (PET) was performed as published elsewhere [21–24]. In brief, BLI (exposure times 1 s, 10 s, and 60 s) of anesthetized mice in prone position was performed using a dedicated small animal multimodal imaging system (In-Vivo Xtreme) 10–12 min after intraperitoneal injection of 200 μl of D-luciferin (15 mg/ml). In parallel, an X-ray image was taken from the same animals at the same position. MRI of continuously anesthetized mice was performed using a 7 T small animal imaging system BioSpin 70/30 (Bruker). Motion artifacts were reduced using a respiratory gating module (SA Instruments, Stony Brook, NY, USA). T2-weighted image series were acquired using the TRARE sequence with an echo time of 38 ms and a repetition time of 4724.9 ms at a resolution of 0.2 × 0.2 × 0.6 mm and an intersection space of 0.8 mm. PET investigations were performed as a pilot experiment in two SCID beige mice using a dedicated small animal PET/CT system (NanoPET/CT, Mediso, Budapest, Hungary). For targeting of EGFR as molecular characteristic of UM-UC-3^LUC^K1 cells engrafted in the bladder the ^68^Ga-radiolabeled EGFR antibody cetuximab (27 MBq; antibody modified with NOTA (1,4,7-triazacyclonane-1,4,7-triacetic acid) as ^68^Ga-chelator) was transurethrally injected. After 30 min incubation and flushing with PBS (0.3 ml) for three times static scan PET acquisition was done at 1 h after administration. Afterwards, transmission CT was acquired. Then the bed-fixed animal was positioned in the MRI system and, in addition, registered T2-weighted image was acquired to get high contrast between the urine with high intensity and the tumor tissue with lower intensity.

## Results

### Bioluminescence characterization of UM-UC-3^LUC^K1 in vitro and in vivo

**Fig. 1:**
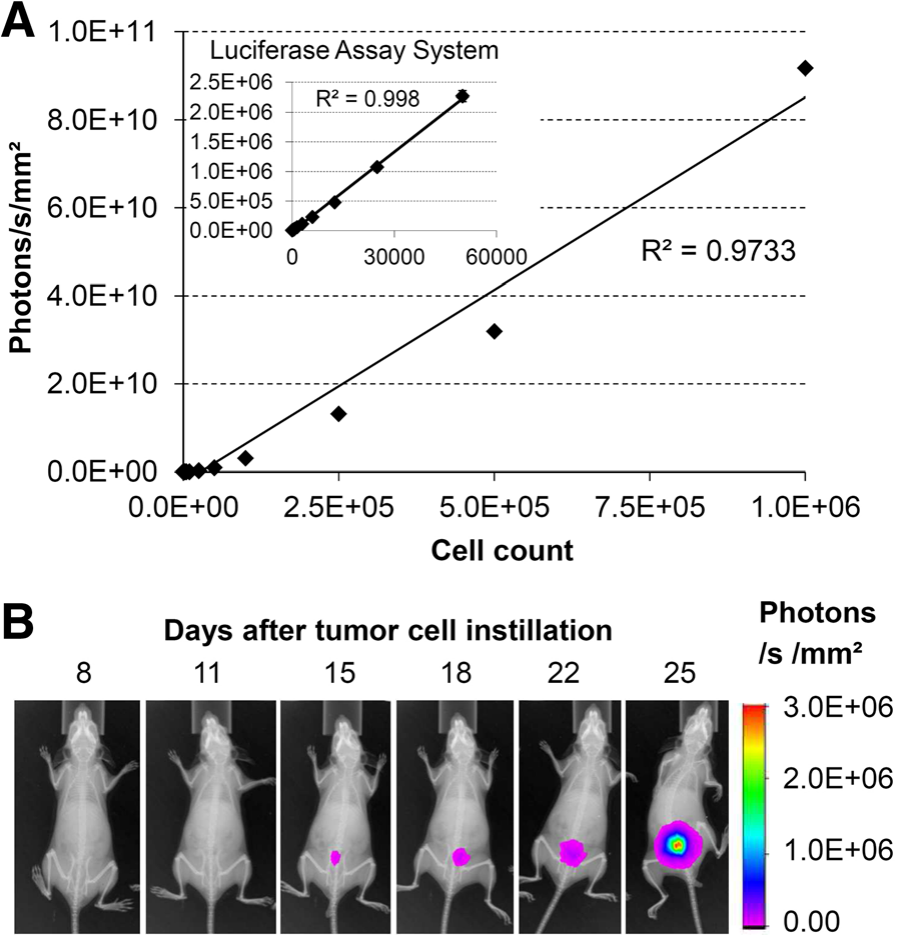
Bioluminescence signal intensities of UM-UC-3^LUC^-K1 cells in vitro in a 96-well plate (**a**) and in vivo after instillation into the bladder of a SCID-beige mouse from experiment 3 (**b**) measured using In-Vivo Xtreme imaging system. The insert in (**a**) shows luciferase signal measured in vitro with the Luciferase Assay System (x-axis: cell count, y-axis: relative light units)

The luciferase expressing UM-UC-3^LUC^K1 clone was generated to enable non-invasive tumor detection in the mouse bladders. Besides periodic measurement of luciferase activity with the Luciferase Assay System, the in vitro luminescence intensity was quantified after D-luciferin incubation using the In-Vivo Xtreme imaging system. A strong relationship of luminescence intensity and cell count was observed with both measuring methods (Fig. 1a). A representative in vivo measurement series is shown in Fig. 1b. This SCID-beige mouse displayed first luciferase signal 15 days after tumor cell instillation. Tumor growth could be monitored for 10 days with steadily rising luminescence intensity. On day 25 the mouse was sacrificed due to high tumor load as indicated by the signal intensity.

### Optimization of orthotopic bladder cancer growth

First, the period of time between harvesting the cells and instillation into the mouse bladder was an important factor for optimal tumor growth. Although >96% of UM-UC-3^LUC^K1 cells were vital 5 h after incubation in culture media, PBS and urine, respectively, no in vivo tumor growth was achieved in NMRI nude mice when the time span between harvesting the cells and instillation was 2 h or longer.

**Fig. 2:**
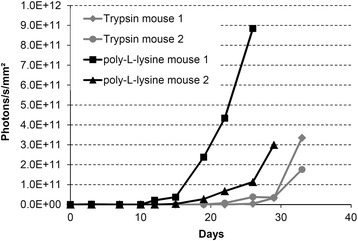
Increase of the luminescence intensity of UM-UC-3^LUC^K1 cells after transurethral instillation of 2.0 × 10^6^ cells into NMRI nude mice for 2 h (experiment 1). Bladder wall was treated with either trypsin or poly-L-lysine before tumor cell inoculation

Based on literature studies, NMRI nude mice were selected for establishing an orthotopic UM-UC-3^LUC^K1 BCa model. However, only 22–40% of NMRI nude mice developed a bladder tumor, although, pretreatment of the urinary bladders was performed before instillation of 2.0 × 10^6^ tumor cells for 2 h in two independent experiments (Table 1, No 1 and 2). There was no difference in tumor cell engraftment comparing the bladder pretreatment with trypsin and PLL (Table 1, No 1). The induction of lesions in the mucosa by carefully scratching with the cannula of the permanent venous catheters did not considerably improve tumor cell engraftment after PLL pretreatment (Table 1, No 2). Therefore, PLL pretreatment – without scratching with the cannula – was selected for further experiments. Exemplarily, the development of BLI signal intensities of the four tumor-bearing NMRI nude mice in experiment 1 is shown in Fig. 2.

A switch in the mouse strain to BALB/c nude and SCID-beige mice increased tumor cell engraftment to 70% and 100%, respectively (Table 1, No 3). Since BALB/c nude mice showed first BLI signal late – after 33 days on average – and with high variance, cell count for tumor cell instillation was increased to 5.0 × 10^6^ in the next experiment. In contrast, tumor cell count was decreased to 1.0 × 10^6^ in SCID-beige mice because of the fast tumor growth that is reflected by the short period of signal duration of 8.7 days on average (Table 1, No 3). The signal duration represents the possible treatment period in the evaluation of new therapeutics and should be at least two weeks.

**Fig. 3:**
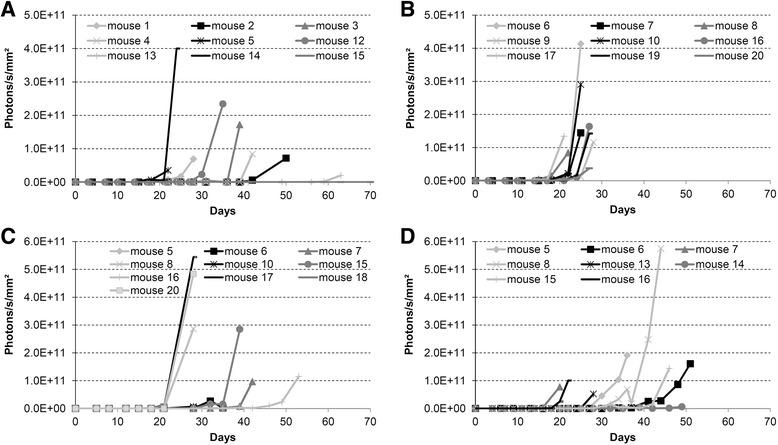
Increase of the bioluminescence signal of UM-UC-3^LUC^K1 cells after transurethral instillation (**a**) of 5.0 × 10^6^ cells into BALB/c nude mice for 2 h (experiment 4A), (**b**) of 1.0 × 10^6^ cells into SCID-beige mice for 2 h (experiment 4B), (**c**) of 1.0 × 10^6^ cells into SCID-beige mice for 1 h (experiment 5B) and (**d**) of 0.5 × 10^6^ cells into SCID-beige mice for 30 min (experiment 6B). Bladder wall was treated with poly-L-lysine before tumor cell inoculation

With the adjusted cell counts the mean time until occurrence of first BLI signal remained at days 33.9 ± 18.3 (mean deviation) for BALB/c nude and at days 14.8 ± 2.1 for SCID-beige mice (Table 1, No 4). The differences in BLI signal intensity development for the individual animals of both mouse strains are shown in Fig. 3a and b. Due to this late onset of tumor growth with its high variance in BALB/c nude mice, further optimization was done using SCID-beige mice. To extend the short mean signal duration of 10.4 days in experiment 4, cancer cell count was further decreased.

Instillation of 1.0 × 10^6^ and 0.5 × 10^6^ cancer cells in SCID-beige mice in combination with a decrease in instillation time to 1 h caused a shift in the start of tumor detection to days 25.8 and 22.4 (mean values), respectively (Table 1, No 5). Exemplarily, BLI signal intensities of mice after instillation of 1.0 × 10^6^ UM-UC-3^LUC^K1 cells are shown in Fig. 3c. The average signal duration remained below two weeks (Table 1, No 5). Therefore, instillation time of UM-UC-3^LUC^K1 BCa cells was further decreased down to 30 min. In this manner, the mean luminescence signal duration extended to 19.6 ± 8.2 days while the mean signal start remained unchanged at 22.4 days (Table 1, No 6).

**Fig. 4:**
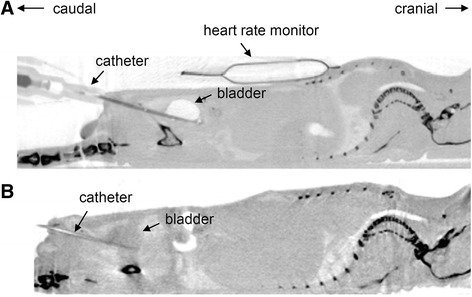
Computed tomograms of BALB/c nude mice during transurethral instillation of tumor cells. Mice are in supine position. A sagittal plane is shown. **a** The mouse was catheterized and tumor cells were injected after connecting the syringe. Because of the air present in the catheter an air bubble is formed in the urinary bladder (visible as bright region). **b** The catheter was filled with tumor cell suspension prior to catheterization and no air bubble is apparent in the bladder

During catheterization of mice bladders an air bubble was formed in the urinary bladder due to the air that was present in the catheter (Fig. 4a). To analyze if this air bubble influences tumor onset, an alternative instillation method was conducted. In doing so, the catheter itself was filled with tumor cell suspension prior to catheterization of the murine bladders. This prevented the air bubble formation (Fig. 4b). The comparison of both instillation techniques showed no differences in tumor formation in SCID-beige mice (Table 1, No 6). Exemplarily, luminescence intensities of individual animals after instillation of 0.5 × 10^6^ UM-UC-3^LUC^K1 cells without the air bubble in the bladder are shown in Fig. 3d.

### Examination of tumors by molecular imaging

**Fig. 5:**
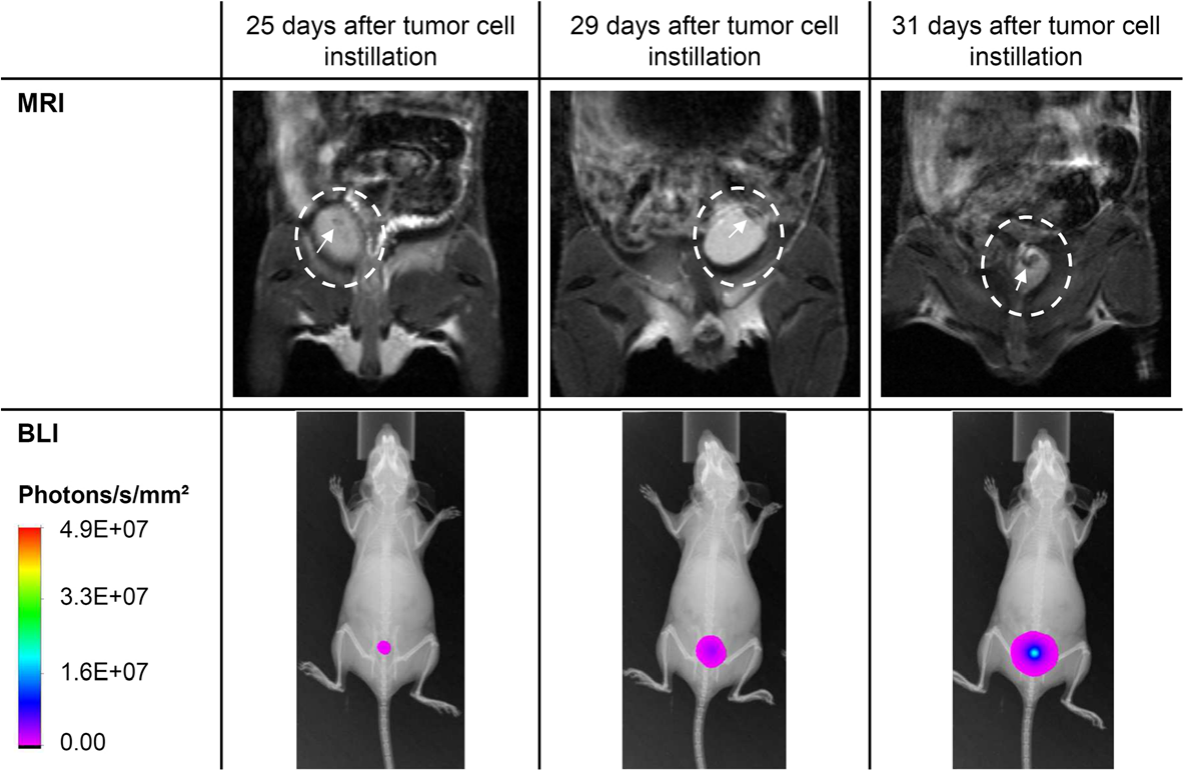
Comparison of MRI and corresponding BLI images of a BALB/c nude mouse from experiment 3 at days 25, 29 and 31 after instillation of 2.0 × 10^6^ UM UC 3^LUC^K1 cells for 2 h. MRI images show a coronal plane. The urinary bladder is marked with a circle and the arrow points at the tumor

Selected animals were analyzed by MRI and PET (combined with CT). MRI measurements were carried out every 2 to 4 days to visualize size, location and growth of the tumor. Exemplarily, the MRI and BLI images of a UM-UC-3^LUC^K1 tumor in a BALB/c nude mouse are shown (Fig. 5). Both imaging techniques displayed the rapid tumor growth within the 6 days shown. In these MRI images, the orthotopic tumor was easily distinguishable in the bladder. Overall, MRI of the urinary bladder of living mice is challenging because of the movement of the bladder and the intestine. Blurring occured preventing the quantitative evaluation in 8 of the 61 imaging series. In 5 cases no tumor could be detected in MRI despite positive BLI signals.

**Fig. 6:**
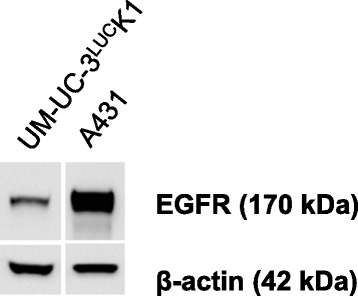
Detection of EGFR protein by Western blotting in UM-UC-3^LUC^K1 BCa cells as well as in A431 epidermoid carcinoma cells that express high levels of EGFR (positive control). Beta-actin was used for loading control

**Fig. 7:**
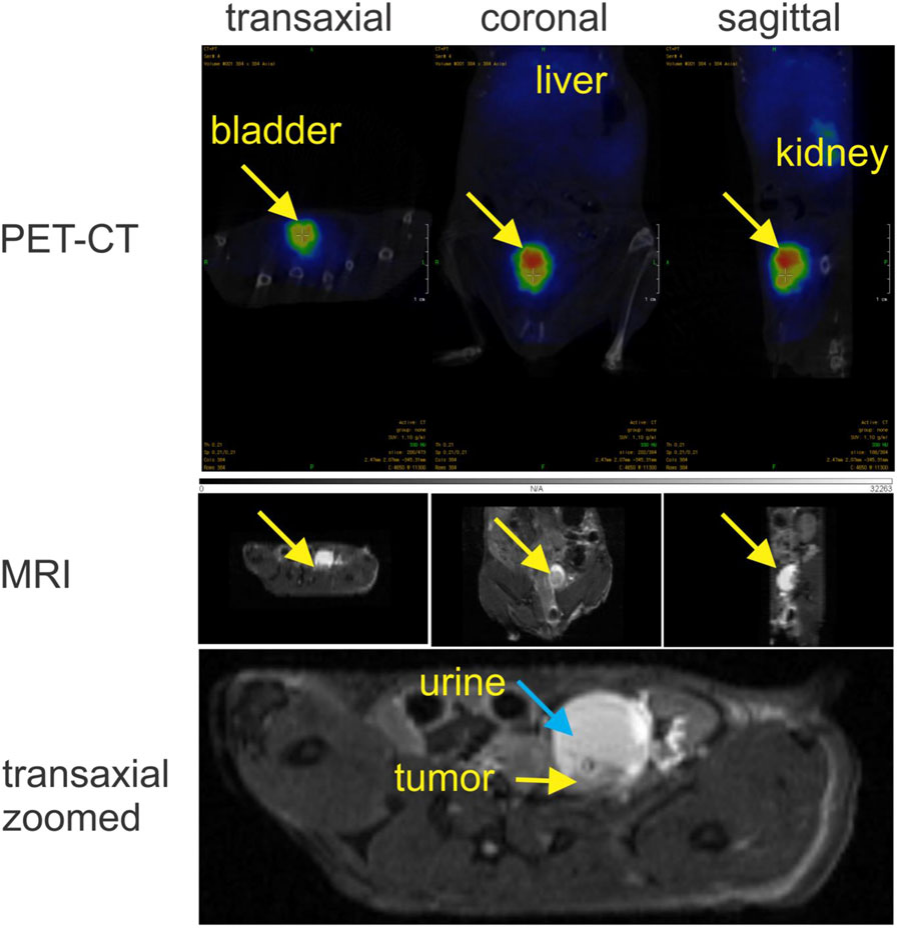
Registered PET (after transurethracally administered ^68^Ga-radiolabeled cetuximab), CT and MRI images (orthogonal sections) of a representative SCID beige mouse. Arrows in the upper and mid panels indicate urinary bladder region. Arrows in the lower differentiate between urine and tumor

Western blot analyses proved presence of EGFR protein in UM-UC-3^LUC^K1 cells (Fig. 6). The pilot PET experiment with the transurethrally administered ^68^Ga-labelled EGFR antibody cetuximab was carried out on two SCID beige mice (Fig. 7). The retaining activity allowed imaging of the bound antibody both in the tumor and the bladder. The registration of the PET and CT images showed the localization of most remaining activity in the tumor region revealing targeting of EGFR-expressing UM-UC-3^LUC^K1 cells.

### Histological characterization of orthotopic UM-UC-3^LUC^K1 xenografts and incidence of metastasis

**Fig. 8:**
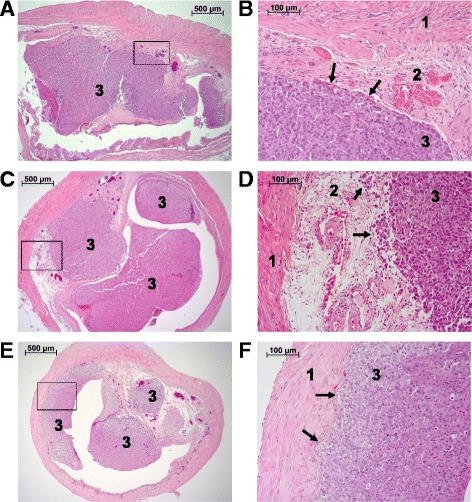
Histological images of UM UC 3LUCK1 xenografts with different TNM classification (**a** and **b**: pTa, **c** and **d**: pT1, **e** and **f**: pT2a). An overview of the bladder and the enlarged section of the box are shown. Arrows point at adjacent tumor tissue. 1 = muscle; 2 = lamina propria; 3 = tumor

**Fig. 9:**
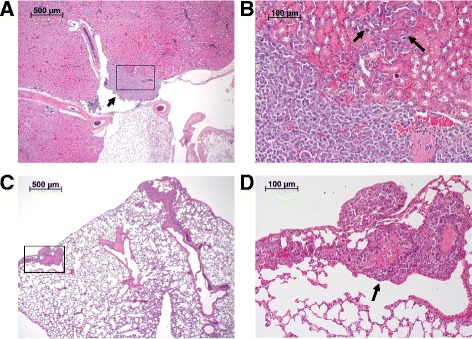
Histological images of renal (**a, b**) and pulmonary (**c, d**) metastasis of orthotopic UM-UC-3^LUC^K1 xenografts in SCID-beige mice. An overview of the tissue and the enlarged section of the box are shown. Arrows point at the tumor tissue

After HE staining, sections of the UM-UC-3^LUC^K1 tumors were examined for staging and grading. Only slices of tumors with association to the urothelium that allowed TNM classification were included in the evaluation. Of the 68 evaluable xenografts 53 (78%) and 11 (16%) displayed tumor stages T1 and Ta, respectively, whereas 4 tumors (6%) already invaded the musculature (pT2a) (Table 2). All muscle invasive tumors were observed in SCID-beige mice. All evaluable tumors were graded as high-grade. Representative histological images are shown in Fig. 8. In 39 cases (57%) a single tumor could be identified in the urinary bladder whereas in 29 cases two or more tumors grew (Table 2). Kidneys, livers and lungs of all 16 mice in experiment 6 were examined histopathologically to evaluate a possible metastasis formation. Two mice with pathological BCa stage Ta and T1, respectively, showed metastasis in the kidneys whereas one of these mice also showed lung metastasis (Fig. 9).

**Table 2:** Histopathological examination of UM-UC-3^LUC^K1 xenografts

Exp No	No of evaluable tumors (total no of tumors)	TNM classification			Grading	No of tumors in urinary bladder	
		pTa	pT1	pT2a		1	> 1
2	NMRI nude: 3 (5)	0 (0%)	3 (100%)	0 (0%)	high grade	1 (33%)	2 (67%)
3	BALB/c nude: 7 (7) SCID-beige: 9 (9)	1 (14%) 1 (11%)	6 (86%) 7 (78%)	0 (0%) 1 (11%)	high grade high grade	4 (57%) 2 (22%)	3 (43%) 7 (78%)
4	BALB/c nude: 9 (9) SCID-beige: 8 (9)	2 (22%) 3 (38%)	7 (78%) 3 (38%)	0 (0%) 2 (25%)	high grade high grade	9 (100%) 3 (38%)	0 (0%) 5 (62%)
5	SCID-beige: 18 (19)	2 (11%)	16 (89%)	0 (0%)	high grade	13 (72%)	5 (28%)
6	SCID-beige: 14 (15)	2 (14%)	11 (79%)	1 (7%)	high grade	7 (50%)	7 (50%)

## Discussion

The evaluation of novel anticancer agents requires suitable animal models to continue research after successful cell culture experiments and before entering clinical trials. Orthotopic animal models with xenogenic human BCa cells closely mimic the natural microenvironment of the human tumor and allow intravesical therapy application as well as studying metastasis formation. However, they do not enable immunological examinations because of the necessity to use immunodeficient animals. Mice are well suited for the establishment of an orthotopic BCa xenograft since the structure and function of their lower urinary tract show great similarities to humans [25]. Because of simple handling during bladder catheterization female mice should be used [25]. For the reliability and reproducibility of the animal model a high rate of tumor cell engraftment is necessary. Tumor growth should be homogeneous in all animals and should offer a suitable treatment period of at least two weeks. Multiple parameters can affect tumor cell engraftment and growth behavior. Most importantly, the tumor cells have to be instilled as soon as possible after harvesting. While UM-UC-3^LUC^K1 engraftment rate decreased when cells were instilled ≥2 h after harvesting, time periods shorter than 20 min and 1 h, respectively, were recommended for breast and prostate cancer cells [26, 27]. Interestingly, the formation of an air bubble in the murine bladder – which occurred if the bladder was catheterized with an empty catheter and tumor cells were instilled thereafter – did not alter tumor cell engraftment.

The immunologic characteristics of the mouse strain have significant impact on tumor development. Orthotopic UM-UC-3^LUC^K1 tumor formation was observed in 22–40% of NMRI nude mice, 70–90% of BALB/c nude and 88–100% of SCID-beige. While all three mouse strains lack T cells, SCID-beige mice also lack B cells and have impaired natural killer cell activity. Therefore, these mice were more susceptible for tumor engraftment. Ye et al. examined the growth of a human adenocarcinoma alveolar basal epithelial cell line (A549) after subcutaneous injection in six immunodeficient mouse strains [28]. A NSI strain (NOD-*scid*-*IL2Rg*−/−) without T, B and natural killer cells was most accessible for tumor growth. Already 1.0 × 10^4^ A549 cells induced a subcutaneous tumor in these mice whereas 1.0 × 10^5^ cancer cells were necessary in SCID, NOD-SCID and nude mice. A tumor engraftment index was developed to quantify the immunodeficiency of the mouse strains [28]. Such an index for all available immunodeficient mouse strains would be very helpful for the expedient selection of a suitable mouse strain for the establishment of heterotopic as well as orthotopic xenograft models. Van der Horst et al. instilled UM-UC-3^LUC2^ cells into BALB/c nude mice and achieved 73% orthotopic tumor cell engraftment which is comparable to our study [11]. The firefly luciferase 2 (LUC2) gene used in the study of van der Horst et al. is codon optimized to improve gene expression in mammalian cells [29]. Its enzyme activity is ten times higher than that of the luciferase LUC+ used in this study. With the use of LUC2, the start of luminescence intensity detection in the present study might have been earlier but it would not have influenced cancer cell engraftment. The research on luciferase genes and substrates is ongoing and will continuously improve BLI, current developments are recently reviewed in [30].

Next, the tumorigenic potential of the cell line is of importance. As we aimed at generating an orthotopic model for high-risk NMIBC and as successful tumor growth was reported for UM-UC-3 cells previously [31] this cell line was chosen for our experiments. However, not all cancer cell lines will form a tumor after implantation in mice. For example, UM-UC-3 cells – but not 5637, 253 J and TCCSUP BCa cells – grew orthotopically in BALB/c nude mice [31]. Furthermore, of 10 cell lines derived from malignant urinary tract neoplasms, two were not tumorigenic in athymic nude mice whereas five cell lines (UM-UC-1, UM-UC-3, UM-UC-6, UM-UC-9 and UM-UC-14) produced subcutaneous tumors with a diameter of 1.0–1.5 cm already on days 9 to 19 after injection of 1.0 × 10^7^ cells [32]. Experiments using KU-7 cells – a popular cell line isolated from a patient with low grade papillary BCa in 1980 which was used in numerous studies for instillation into the bladder – should be considered in the knowledge that these cells were cross contaminated with the cervical carcinoma cell line HeLa before 1984 at the source institution [33]. Therefore, a careful selection of cell lines is necessary.

To facilitate orthotopic tumor formation it is necessary to overcome the glycosaminoglycan layer of the bladder mucosa either mechanically or chemically (reviewed in [8, 9]). Briefly, initial approaches using open surgical procedures as well as bladder pretreatment with hydrochloric acid or silver nitrate resulted in health complications for the animals. Pretreatment with either trypsin (a serine protease) or PLL (a cationic polypeptide enhancing the electrostatic interaction between the bladder mucosa and the cancer cells), respectively, represent more gentle procedures and were therefore applied in the present study. The rupture of the mucosa with a stylet can facilitate tumor engraftment as it was shown in orthotopic homo- and xenograft BCa models in mice [34]. However, there is the danger of bladder perforation by the cannula. Since we observed no difference in tumor cell engraftment after trypsin or PLL pretreatment and scratching with the cannula of the permanent venous catheters did not significantly enhance tumor engraftment, the gentlest pretreatment – PLL without scratching – was chosen for further optimization.

In previous studies, cell count for transurethral instillation of xenogenic BCa cells varied between 2.0 × 10^6^ and 1.0 × 10^7^ cells in an injection volume of 35–100 μl [10–13, 31, 34]. Generally, the dwell time of tumor cells in the murine bladder has been two to three hours and tumor engraftment rates of 67–100% have been achieved after mechanical or chemical bladder pretreatment [10–13, 31, 34]. In none of these studies a variation of any parameter that might influence tumor growth has been reported. For orthotopic growing UM-UC-3^LUC^K1 cells in BALB/c nude mice an enhancement of the tumor engraftment rate was achieved in our study by increasing cell count. Furthermore, the luminescence signal duration – which characterizes the possible treatment period – could be modified by changing the tumor cell dwell time in SCID-beige mice. The most reliable UM-UC-3^LUC^K1 xenograft model was achieved after bladder pretreatment with PLL and instillation of 1.0 × 10^6^ cells for 2 h in SCID-beige mice. In doing so a high rate of tumor engraftment of 100% and an appropriate start of luminescence intensity detection in the bladder – approximately 15 days after tumor cell instillation – were observed. All these xenografts grew comparable. A minor disadvantage of this model is the fast tumor growth with a mean luminescence signal duration of 10.4 days only which offers a treatment period <2 weeks.

In individual cases, transurethrally instilled UM-UC-3^LUC^K1 grew invasively into the bladder muscle (4 of 68 mice) or formed distant metastasis (2 of 16 SCID-beige mice; NMRI nude and BALB/c nude mice were not analyzed for metastasis). This is in accordance with the findings on UM-UC-3^LUC2^ cells in Balb/c nude mice in the study of van der Horst et al., whereas there is no information regarding the frequency of occurrence in their study [11]. It has to be noted that in our study muscle invasive UM-UC-3^LUC^K1 xenografts were found only in the SCID-beige mouse strain which exhibits the highest level of immunodeficiency. Since the SCID-beige mouse with renal and pulmonary metastases had a BCa with tumor stage Ta – which usually does not metastasize – it can not be excluded that metastasis formation is caused as a result of the instillation technique meaning that the instillation volume of 100 μl may have induced an overdistension of the bladder and in consequence a vesicorenal reflux as discussed by Hadaschik et al. [35]. Apparently, cancer cells have been distributed from the kidneys to the lungs via the bloodstream. Therefore, this mouse rather has a pT3 tumor of the kidney than a renal metastasis of the Ta tumor. However, van der Horst et al. observed lung metastasis even after instillation of UM-UC-3^LUC2^ cells in a small suspension volume of 35 μl – whereby the dwell time was 3 h. Further evaluation of the metastasis formation of transurethrally injected UM-UC-3 cells is necessary. In doing so, the instillation volume and dwell time should be as low as possible.

BLI is a sensitive, easy handling and relatively high throughput, fast and inexpensive technique for non-invasive monitoring of intravesical growth of luciferase-expressing cancer cells [6]. MRI enables high spatial resolution, but has low sensitivity and throughput as well as high costs [6]. Because of the movement of the intestine, MRI of the bladder of living mice is challenging. However, a distinct linear relationship (R^2^ = 0.929) between luminescence intensity and tumor volume has been shown by MRI on explanted bladders which is not compromised by motion artifacts [35]. In our study both imaging techniques were used to complement each other. While BLI was best for routine measurements, MRI gave information regarding tumor size and location. Attention has to be paid if the tumors evolve large hypoxic and necrotic areas because this reduces luminescence intensities [36]. In MRI flat tumors might be overlooked especially if the bladder is stretched because of high filling. Therefore, a combination of different imaging methods such as BLI plus MRI or BLI plus high resolution ultrasound plus photo-acoustic imaging might give a more complete picture of orthotopic BCa growth [37]. The pilot experiment with ^68^Ga-radiolabeled cetuximab allowed for identification of engrafted EGFR-expressing tumor cells in the bladder, and, furthermore, demonstrated the principal usability of radioimmunologic diagnostics of such tumors in the bladder. Functional characterization of EGFR expression in BCa, on the other hand, is a prerequisite for personalized targeted local treatment with radionuclide-based [38, 39] or immunologic [21] approaches.

## Conclusions

With the optimized protocol in SCID-beige mice – bladder pretreatment with poly-L-lysine, transurethral instillation of 1.0 × 10^6^ UM-UC-3^LUC^K1 bladder cancer cells for 2 h – an applicable and reliable model for high-risk non-muscle invasive bladder cancer was achieved. The model will be used for the development of theranostic approaches, particularly, by local application in the bladder using PET, radioimmunologic and retargeting strategies.

## Abbreviations

BCa: Bladder cancer
BLI: Bioluminescence imaging
EGFR: Epidermal growth factor receptor
MRI: Magnetic resonance imaging
NMIBC: Non-muscle invasive bladder cancer
PET: Positron emission tomography
PLL: Poly-L-lysine

## References

[1] Ferlay J, Soerjomataram I, Dikshit R, Eser S, Mathers C, Rebelo M (2015). Cancer incidence and mortality worldwide: sources, methods and major patterns in GLOBOCAN 2012. Int J Cancer.

[2] Cancer in Germany 2011/2012. vol. 10th edition. Berlin: Robert Koch Institute (ed.) and the Association of Population-based Cancer Registries in Germany (ed). 2016.

[3] Babjuk M, Bohle A, Burger M, Capoun O, Cohen D, Comperat EM (2017). EAU guidelines on non-muscle-invasive Urothelial carcinoma of the bladder: update 2016. Eur Urol.

[4] van den Bosch S, Alfred Witjes J (2011). Long-term cancer-specific survival in patients with high-risk, non-muscle-invasive bladder cancer and tumour progression: a systematic review. Eur Urol.

[5] Kuntner C, Stout D (2014). Quantitative preclinical PET imaging: opportunities and challenges. Front Phys.

[6] Lyons SK (2015). Imaging mouse models of cancer. Cancer J.

[7] Ullrich M, Bergmann R, Peitzsch M, Zenker EF, Cartellieri M, Bachmann M (2016). Multimodal Somatostatin receptor Theranostics using [(64)cu]cu−/[(177)Lu]Lu-DOTA-(Tyr(3))octreotate and AN-238 in a mouse Pheochromocytoma model. Theranostics..

[8] Chan E, Patel A, Heston W, Larchian W (2009). Mouse orthotopic models for bladder cancer research. BJU Int.

[9] Zhang N, Li D, Shao J, Wang X (2015). Animal models for bladder cancer: the model establishment and evaluation (review). Oncol Lett.

[10] Tanaka M, Gee JR, De La Cerda J, Rosser CJ, Zhou JH, Benedict WF (2003). Noninvasive detection of bladder cancer in an orthotopic murine model with green fluorescence protein cytology. J Urol.

[11] van der Horst G, van Asten JJ, Figdor A, van den Hoogen C, Cheung H, Bevers RF (2011). Real-time cancer cell tracking by bioluminescence in a preclinical model of human bladder cancer growth and metastasis. Eur Urol.

[12] Watanabe T, Shinohara N, Sazawa A, Harabayashi T, Ogiso Y, Koyanagi T (2000). An improved intravesical model using human bladder cancer cell lines to optimize gene and other therapies. Cancer Gene Ther.

[13] Pfost B, Seidl C, Autenrieth M, Saur D, Bruchertseifer F, Morgenstern A (2009). Intravesical alpha-radioimmunotherapy with 213Bi-anti-EGFR-mAb defeats human bladder carcinoma in xenografted nude mice. J Nucl Med.

[14] Jager W, Moskalev I, Janssen C, Hayashi T, Awrey S, Gust KM (2013). Ultrasound-guided intramural inoculation of orthotopic bladder cancer xenografts: a novel high-precision approach. PLoS One.

[15] Becker MN, KJ W, Marlow LA, Kreinest PA, Vonroemeling CA, Copland JA (2014). The combination of an mTORc1/TORc2 inhibitor with lapatinib is synergistic in bladder cancer in vitro. Urol Oncol.

[16] Sherf BA, Wood KV (1994). Firefly luciferase engineered for improved genetic reporting. Promega Notes Magazin.

[17] Tietze S, Schau I, Michen S, Ennen F, Janke A, Schackert G et al. A Poly(Propyleneimine) Dendrimer-Based Polyplex-System for Single-Chain Antibody-Mediated Targeted Delivery and Cellular Uptake of SiRNA. Small. 2017;13(27):1700072. 10.1002/smll.201700072 28544767

[18] Temme A, Rieger M, Reber F, Lindemann D, Weigle B, Diestelkoetter-Bachert P (2003). Localization, dynamics, and function of survivin revealed by expression of functional survivinDsRed fusion proteins in the living cell. Mol Biol Cell.

[19] Erdmann K, Kaulke K, Rieger C, Salomo K, Wirth MP, Fuessel S (2016). MiR-26a and miR-138 block the G1/S transition by targeting the cell cycle regulating network in prostate cancer cells. J Cancer Res Clin Oncol.

[20] Sobin LH, Gospodarowicz MK, Wittekind C. TNM Classification of Malignant Tumours. 7th ed. Oxford: Wiley-Blackwell; 2011.

[21] Albert S, Arndt C, Feldmann A, Bergmann R, Bachmann D, Koristka S (2017). A novel nanobody-based target module for retargeting of T lymphocytes to EGFR-expressing cancer cells via the modular UniCAR platform. Oncoimmunology.

[22] Feldmann A, Arndt C, Bergmann R, Loff S, Cartellieri M, Bachmann D (2017). Retargeting of T lymphocytes to PSCA- or PSMA positive prostate cancer cells using the novel modular chimeric antigen receptor platform technology “UniCAR”. Oncotarget.

[23] Schubert M, Bergmann R, Forster C, Sihver W, Vonhoff S, Klussmann S (2017). Novel tumor Pretargeting system based on complementary l-configured Oligonucleotides. Bioconjug Chem.

[24] Tondera C, Hauser S, Kruger-Genge A, Jung F, Neffe AT, Lendlein A (2016). Gelatin-based Hydrogel degradation and tissue interaction in vivo: insights from multimodal preclinical imaging in Immunocompetent nude mice. Theranostics.

[25] Reis LO, Sopena JM, Favaro WJ, Martin MC, Simao AF, Reis RB (2011). Anatomical features of the urethra and urinary bladder catheterization in female mice and rats. An essential translational tool. Acta Cir Bras.

[26] Bailey-Downs LC, Thorpe JE, Disch BC, Bastian A, Hauser PJ, Farasyn T (2014). Development and characterization of a preclinical model of breast cancer lung micrometastatic to macrometastatic progression. PLoS One.

[27] Park SI, Kim SJ, McCauley LK, Gallick GE (2010). Pre-clinical mouse models of human prostate cancer and their utility in drug discovery. Curr Protoc Pharmacol.

[28] Ye W, Jiang Z, Li GX, Xiao Y, Lin S, Lai Y (2015). Quantitative evaluation of the immunodeficiency of a mouse strain by tumor engraftments. J Hematol Oncol.

[29] Gambhir SS, Yaghoubi SS. Molecular Imaging with Reporter Genes. vol. 1. Cambridge: Cambridge University Press; 2010.

[30] Mezzanotte L, van’t Root M, Karatas H, Goun EA, Lowik C (2017). In vivo molecular bioluminescence imaging: new tools and applications. Trends Biotechnol.

[31] Nogawa M, Yuasa T, Kimura S, Tanaka M, Kuroda J, Sato K (2005). Intravesical administration of small interfering RNA targeting PLK-1 successfully prevents the growth of bladder cancer. J Clin Invest.

[32] Sabichi A, Keyhani A, Tanaka N, Delacerda J, Lee IL, Zou C (2006). Characterization of a panel of cell lines derived from urothelial neoplasms: genetic alterations, growth in vivo and the relationship of adenoviral mediated gene transfer to coxsackie adenovirus receptor expression. J Urol.

[33] Jager W, Horiguchi Y, Shah J, Hayashi T, Awrey S, Gust KM (2013). Hiding in plain view: genetic profiling reveals decades old cross contamination of bladder cancer cell line KU7 with HeLa. J Urol.

[34] Yang XH, Ren LS, Wang GP, Zhao LL, Zhang H, Mi ZG (2012). A new method of establishing orthotopic bladder transplantable tumor in mice. Cancer Biol Med.

[35] Hadaschik BA, Black PC, Sea JC, Metwalli AR, Fazli L, Dinney CP (2007). A validated mouse model for orthotopic bladder cancer using transurethral tumour inoculation and bioluminescence imaging. BJU Int.

[36] Black PC, Shetty A, Brown GA, Esparza-Coss E, Metwalli AR, Agarwal PK (2010). Validating bladder cancer xenograft bioluminescence with magnetic resonance imaging: the significance of hypoxia and necrosis. BJU Int.

[37] Scheepbouwer C, Meyer S, Burggraaf MJ, Jose J, Molthoff CF (2016). A multimodal imaging approach for longitudinal evaluation of bladder tumor development in an Orthotopic Murine model. PLoS One.

[38] Koi L, Bergmann R, Bruchner K, Pietzsch J, Pietzsch HJ, Krause M (2014). Radiolabeled anti-EGFR-antibody improves local tumor control after external beam radiotherapy and offers theragnostic potential. Radiother Oncol.

[39] Sihver W, Pietzsch J, Krause M, Baumann M, Steinbach J, Pietzsch HJ (2014). Radiolabeled Cetuximab conjugates for EGFR targeted cancer diagnostics and therapy. Pharmaceuticals (Basel).

